# Recent advances in NIR-II photothermal and photodynamic therapies for drug-resistant wound infections

**DOI:** 10.1016/j.mtbio.2025.101871

**Published:** 2025-05-14

**Authors:** Xiang Chen, Zhanming Lin, Nuo Cheng, Yongjun Mo, Liang Lu, Jun Hou, Zhenghui Li, Xinyu Nie, Shuai Gao, Qikai Hua

**Affiliations:** a Department of Bone and Joint Surgery (Guangxi Diabetic Foot Salvage Engineering Research Center/Research Centre for Regenerative Medicine), The First Affiliated Hospital of Guangxi Medical University, 530021, Nanning, China; b Department of Gastroenterology, Shanghai Fifth People's Hospital, Fudan University, 200240, Shanghai, China; c Department of Orthopaedics, Guigang City People's Hospital, 537100, Guigang, China; d Department of Orthopedics, The First Affiliated Hospital of USTC, Division of Life Sciences and Medicine, University of Science and Technology of China, Hefei, 230002, China; e Collaborative Innovation Centre of Regenerative Medicine and Medical Bio-Resource Development and Application Co-constructed by the Province and Ministry, Guangxi Medical University, Nanning, Guangxi, 530021, China; f Department of Neurosurgery, The Third Affiliated Hospital of Zhengzhou University, Zhengzhou University, Zhengzhou, Henan, 450052, China; g Harvey Cushing Neuro-Oncology Laboratories, Department of Neurosurgery, Brigham and Women's Hospital, Harvard Medical School, Boston, MA, 02115, USA; h Department of Chemistry, Massachusetts Institute of Technology, Cambridge, MA, 02139, USA

**Keywords:** Near-infrared II, Photothermal therapy, Photodynamic therapy, Wound infections, Wound healing

## Abstract

Bacterial infection can delay wound healing, while drug resistance further complicates the treatment of wound infection. Phototherapy, including photothermal therapy (PTT) and photodynamic therapy (PDT), is a non/mini-invasive and efficient antibacterial strategy that rarely induces bacterial resistance. This treatment relies on specific wavelengths of light to activate photothermal agents (PTAs) or photosensitizers, killing bacteria by generating local heats or reactive oxygen species (ROS), respectively. However, the light for traditional PTT/PDT mainly falls in the visible and near-infrared I light (Vis/NIR-I light, 400–900 nm) regions, which significantly limits further clinical translations due to its low tissue permeability. The near-infrared II (NIR-II,1000–1700 nm) light is increasingly utilized in antibacterial PTT/PDT to improve tissue penetration and ameliorate the immune microenvironment of deeper wounds. Meanwhile, NIR-II light offers a higher maximum permissible exposure (MPE) for PTT/PDT in treating wound infections, thereby facilitating the security, in comparison to Vis/NIR-I light. This review highlights recent advancements in NIR-II PTT/PDT for drug-resistant wound infections, focusing on mechanisms, therapeutic outcomes, challenges, and prospects.

## Graphical abstract

**Figure:**
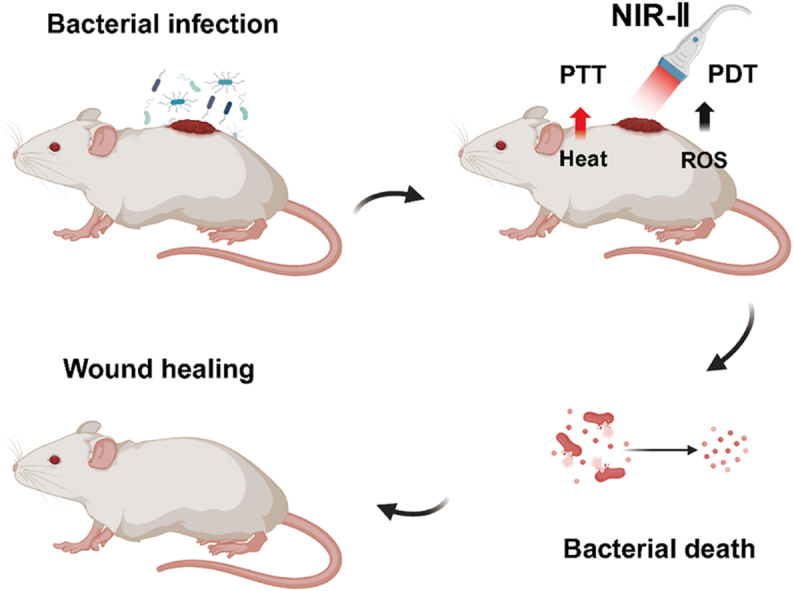
Schematic representation of NIR-II photothermal therapy (PTT) and photodynamic therapy (PDT) for treating drug-resistant wound infections.

## 1 Introduction

**Fig. 1:**
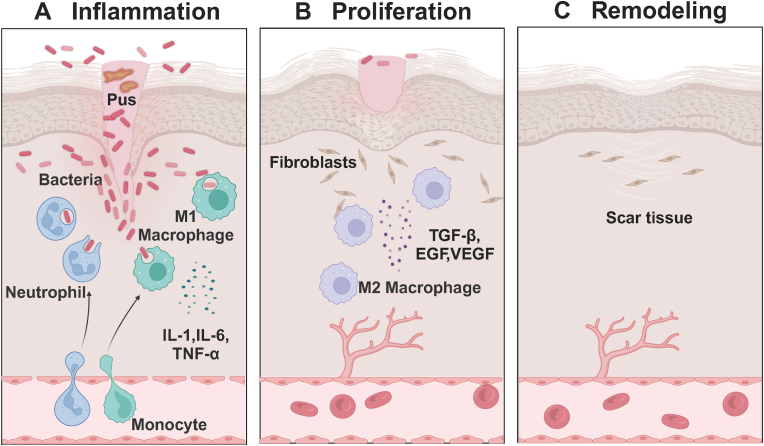
Schematic illustration of the process of infected wound healing. (A) Inflammation. The wound becomes infected with bacteria, and proinflammatory macrophages (M1) produce proinflammatory factors such as interleukin-1 (IL-1), interleukin-6 (IL-6), and tumor necrosis factor-α (TNF-α). Neutrophils are recruited to remove bacteria and produce pus. (B) Proliferation. Macrophages transition to M2 type and release healing cytokines such as transforming growth factor β (TGF-β), epidermal growth factor (EGF), vascular endothelial growth factor (VEGF), accompanying granulation tissue formation, angiogenesis, and epithelial regeneration. (C) Remodeling. Cells rearrange, and disorganized collagen is degraded and absorbed. Created with BioRender.com.

Skin injury results in wound formation, which compromises the skin's immune and protective functions, making the site vulnerable to bacterial wound infections, which in turn delay the healing process [1,2]. The healing of wounds involves three phases: inflammation, proliferation, and remodeling, each essential for transitioning from infection to complete recovery. During the early inflammatory stage, pro-inflammatory macrophages (M1) produce pro-inflammatory factors, such as Interleukin-1 (IL-1), Interleukin-6 (IL-6), and Tumor Necrosis Factor-alpha (TNF-α). Neutrophils are recruited to clear bacteria, and subsequently, macrophages gradually transform to the anti-inflammatory type (M2), progressing to the proliferation stage [3,4]. The proliferation stage is marked by the migration of fibroblasts, along with granulation, angiogenesis, and epithelial regeneration. During the remodeling stage, type I collagen gradually replaces type III collagen in granulation tissue to improve skin elasticity [5]. Collagen fibers reorganize and rearrange, forming scar tissue, restoring tissue structure, and enhancing strength [3,6,7]. Fig. 1 illustrates the process of infected wound healing.

Due to skin barrier disruption, wounds are susceptible to bacterial infections during the healing process [8]. Antibiotics are essential for treating infected wounds, but overuse can lead to the development of antibiotic-resistant bacteria [9,10]. Infections caused by drug-resistant bacteria create a unique microenvironment at the wound site, which includes neutrophil and macrophage recruitment, abnormal pH, hypoxia, and biofilm formation. These factors further inhibit angiogenesis and tissue regeneration, hindering wound healing [11]. The mechanisms of bacterial resistance include: (1) changes in membrane permeability, which is the primary cause of bacterial resistance. The influx of antibiotics is mainly controlled by porin channels located on the outer membrane, and alterations in porin expression levels or phenotypes can block antibiotic entry; (2) enzymatic degradation, bacteria produce *β*-lactamases to degrade antibiotics such as imipenem, rendering them inactive; (3) DNA mutations, leading to resistance against specific antibiotics; (4) efflux pumps, which are active transport channels in the cell membrane that can expel antibiotics from the cell [12,13]. It is important to note that drug-resistant bacteria often form biofilms, which are encased in a sugar matrix composed of proteins and extracellular DNA. The polysaccharide shell protects the biofilm, making it difficult to remove. Additionally, the biofilm shields drug-resistant bacteria from both antibiotics and the immune system, further complicating the treatment [10,14,15].

In addition to antibiotics, several new methods are currently being explored to treat drug-resistant bacterial infections in wounds. Antimicrobial peptides (AMPs) are a class of drugs, typically fewer than 100 amino acids in length, that are widely distributed in nature. They exhibit broad-spectrum antibacterial activity, primarily targeting bacterial membranes, making them effective in killing drug-resistant bacteria [16,17]. However, the high cost and structural instability of AMPs limit their widespread use [18]. Modern wound dressings are also used in wound treatment. For example, alginate, an anionic polysaccharide derived from brown algae or kelp, is known for its low toxicity and excellent biocompatibility. Due to its fibrous matrix, bacteria may become trapped in alginate, preventing further penetration into the body [19]. However, alginate may leave fibers in the wound, causing inflammation and hindering wound healing [20]. Additionally, some dressings can maintain a moist environment conducive to healing, this same condition can also promote bacterial growth [19]. Debridement, which removes infected tissue, effectively reduces bacterial load in the wound. However, if debridement is incomplete, bacteria can rapidly proliferate, reducing effectiveness and causing side effects such as pain and localized damage, making it unsuitable for repeated use [10,21,22]. Therefore, there is a need to develop new approaches for treating drug-resistant bacterial infections in wounds.

In recent years, phototherapy, including photothermal therapy (PTT) and photodynamic therapy (PDT), has been used to treat bacterial infections. The principle of PTT involves the use of PTAs that generate heat upon light exposure, leading to bacterial cell membrane rupture, protein denaturation, and irreversible bacterial damage [23]. Similarly, PDT works by using photosensitizers that produce ROS upon light exposure, including hydroxyl radicals (·OH), hydrogen peroxide (H_2_O_2_), superoxide (O_2_·-), and singlet oxygen (^1^O_2_), which damage bacterial cell membranes and their internal components, resulting in bacterial killing [24,25]. In summary, phototherapy can disrupt the bacterial cell membrane structure and also damage the structural basis of bacterial resistance, thereby killing drug-resistant bacteria. Additionally, it can degrade biofilms and eliminate drug-resistant bacteria within biofilms [23]. Notably, because the treatment time for phototherapy is short, it rarely leads to the development of new drug-resistant bacteria [26]. Beyond antibacterial effects, PTT/PDT can inhibit the activity of the NF-κB pathway, promoting the conversion of M1 macrophages to M2 macrophages. This, in turn, upregulates VEGF, TGF-β, and interleukin-10 (IL-10) while downregulating IL-6 and TNF-α, thus promoting inflammation resolution, angiogenesis, and wound healing [[27], [28], [29]]. Furthermore, the mild heat generated by PTT enhances blood flow and enzyme activity, activating cellular signaling pathways and increasing the expression of heat shock proteins (HSPs). This further stimulates the immune system, contributing to bacterial killing and promoting wound healing. ROS generated by PDT can induce macrophage autophagy via upregulation of interleukin-1β (IL-1β) and enhance the innate immune response triggered by Toll-like receptors (TLR), thereby boosting macrophage bactericidal activity and tissue repair capabilities [30,31].

**Fig. 2:**
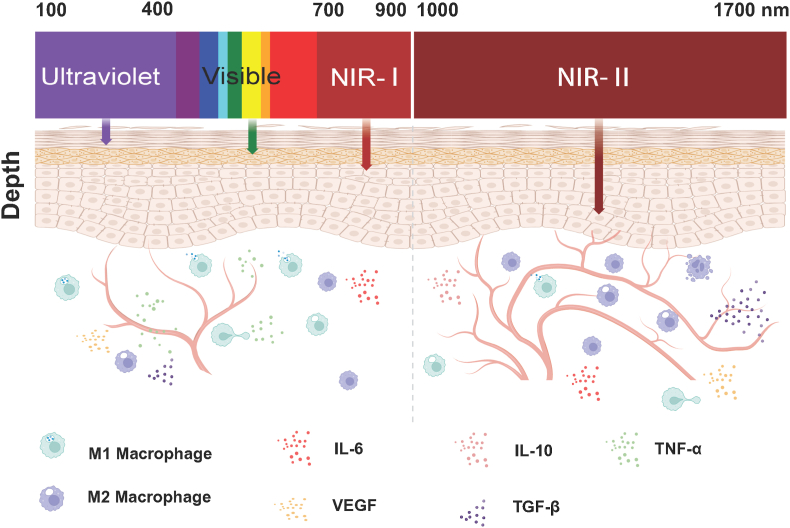
Wavelength ranges and tissue penetration depth of Ultraviolet, Visible light, and near-infrared I (NIR-I) and near-infrared II (NIR-II).

**Fig. 3:**
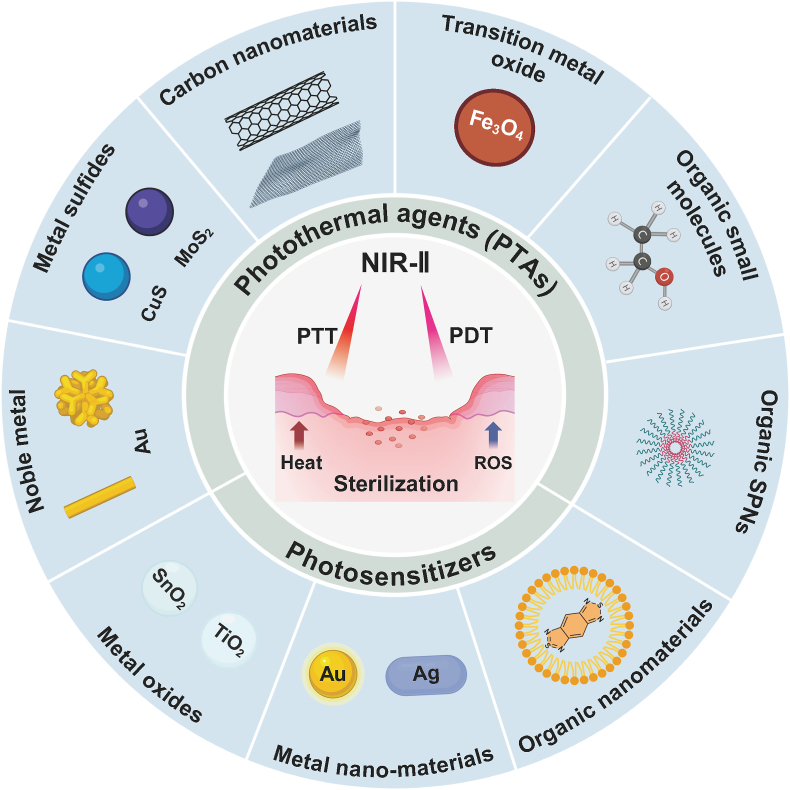
NIR-II photothermal therapy and photodynamic therapy in wound infections by bacteria.

Light sources utilized in phototherapy typically range from ultraviolet–visible light (UV–Vis light, 400–700 nm) to near-infrared (NIR, 700–1700 nm) windows. Fig. 2 illustrates the wavelength ranges and tissue penetration depth of ultraviolet, visible light, and NIR. Among these, NIR light, with its enhanced penetration and diminished side effects compared to shorter wavelengths, enjoys broader applicability [32,33]. In general, NIR can be further divided into two main regions based on wavelength, near-infrared I (NIR-I, 700–900 nm) and near-infrared II (NIR-II, 1000–1700 nm) [34]. Especially, light in the NIR-II region possesses enhanced tissue penetration due to its lower absorption and scattering in biological tissues, paving a new way for treating deep wound infections by bacteria [35]. Besides, NIR-II phototherapy has a higher maximum permissible exposure (MPE), allowing for greater power during illumination without causing tissue damage, thereby providing more effective and safer treatment options compared to NIR-I PTT/PDT [36]. Herein, we summarize the recent application of NIR-II phototherapy in wound infections by bacteria, particularly the treatment of drug-resistant strains (Fig. 3).

## 2 NIR-II PTT for wound infections

### 2.1 History of photothermal therapy

The concept of phototherapy can be dated back to practices in ancient Egypt and Greece, where sunlight was utilized to treat certain illnesses, marking an early form of PTT [37]. The modern PTT began in the 1950s, initially focusing on the direct heating effects of lasers, predominantly for treating skin diseases [38]. The 21st century has evidenced significant achievements of PTT, characterized by the diversification of photothermal conversion efficiency (PCE). Nanotechnology further expands the applications of PTT, with extensive research conducted on nanomaterials due to their unique physicochemical properties and superior PCE. Materials such as gold nanoparticles (Au NPs), carbon nanotubes, and molybdenum disulfide (MoS_2_) have shown promising applications in PTT [36,39,40]. Besides the utilization in tumor treatment and anti-inflammation treatment [[41], [42], [43]], PTT has demonstrated the therapeutic potential for eradicating drug-resistant bacteria, subsequently being an effective supplement to traditional antibiotic treatments [44]. Recently, researchers have been exploring the potential of NIR-II PTT, which offers deeper tissue penetration and lower absorption in biological tissues compared to traditional NIR-I PTT, to treat wound infections of deeper tissue [36].

Additionally, Chen et al. analyzed mouse wound samples 8 days after NIR-II PTT treatment and found that it significantly reduced the levels of four key pro-inflammatory cytokines—TNF-α, IL-6, interleukin-17A (IL-17A) and interferon-beta (IFN-β), and significantly increased the expression of six proteins that promote wound healing, including alpha-smooth muscle actin (α-SMA), collagen type III, vascular endothelial growth factor A (VEGF-A), keratinocyte growth factor (KGF), EGF and fibroblast growth factor-2 (FGF-2, angiogenesis). The experiment demonstrated that the nanomaterials used had high biocompatibility and safety [45]. These findings indicate that NIR-II PTT can reduce inflammation and facilitate wound healing.

### 2.2 Mechanism of photothermal therapy

PTT relies on the conversion of light into heat to kill bacteria. When specific light irradiates PTAs, they will be activated, causing a sharp increase in local temperature to achieve bactericidal clearance. Heat can affect the cell membrane, peptidoglycan, cell wall, RNA, ribosomes, proteins, and the activity of various enzymes [46]. The cell membrane, composed mainly of proteins and lipopolysaccharides, is the fundamental structure that serves as a crucial barrier controlling the entry and exit of substances in bacteria. Damage to the membrane disrupts the respiratory chain, depolarizes the membrane, and impairs heat-sensitive proteins, preventing glucose utilization for energy metabolism [47]. This also leads to the loss of pH homeostasis [48], the leakage of cellular contents, and ultimately bacterial death [[49], [50], [51]]. Specifically, when the local temperature reaches 41–46 °C, cell membrane integrity can be disrupted, leading to dysfunction and thus bacterial death [52,53]. For example, Lin et al. synthesized a hydrogel (CHFH) that achieved local PTT sterilization at 45 °C without damaging surrounding healthy tissues, which was shown to kill 98.2 % of *S. aureus* in an *in vivo* mouse wound model [54]. Additionally, there is a synergistic effect of the combination of PTT and antibiotics. After the induced damage of PTT to the cell membrane, antibiotics can more easily enter the bacteria, thereby accelerating bacterial death [55]. Gao et al. synthesized an injectable hydrogel that carries ciprofloxacin (Cip, an effective antibiotic), which was activated by NIR light to produce the PTT effect, destroying bacterial membranes, while the antibiotic was precisely released at the site of infection, achieving a more effective elimination of bacteria [56].

**Fig. 4:**
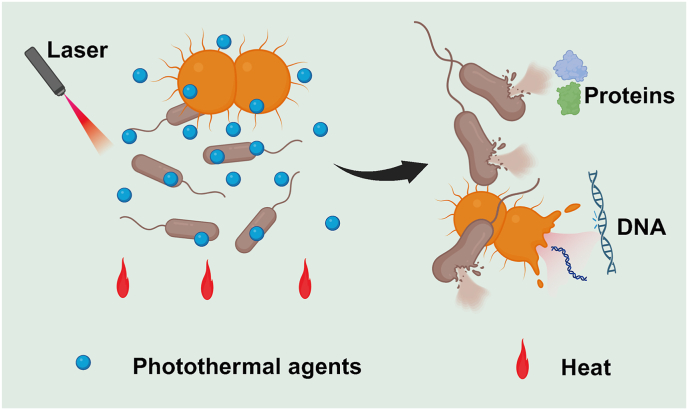
Schematic illustration of the mechanism of antibacterial photothermal therapy (PTT).

In addition to cell membrane damage, exposure of intramembrane structures to high heat can lead to irreversible DNA damage, denaturation and aggregation of proteins within the cell, and inactivation of various enzymes (deoxyribonuclease, DNA repair enzyme, protease, and superoxide dismutase) [57]. Additionally, PTT can stimulate peripheral nerves and trigger neural and humoral reflexes, recruiting more immune cells, including neutrophils and macrophages, to enhance the body's immune defense against bacterial infections. PTT also improves local blood circulation, promotes fibroblast proliferation and collagen synthesis, and enhances tissue repair and regeneration, thereby accelerating wound healing [58]. Ray et al. reported that rapidly rising local temperature can destroy target pathogens through various thermal effects, including denaturation of proteins and enzymes, induction of heat shock proteins, interruption of metabolic signals, swelling of endothelial cells, and formation of microthrombi [59]. Fig. 4 shows the basic mechanism of the antibacterial action of PTT.

### 2.3 Photothermal agents in NIR-II photothermal therapy

PTAs used in PTT can be categorized into two major types: inorganic and organic materials. Inorganic materials contain noble metal ions, metal sulfides, carbon-based nanotubes, and transition metal oxides, while organic PTAs include organic small molecules and semiconductor polymers.

Conventional PTAs irradiated by UV–Vis or NIR-I light are always limited due to their poor tissue penetration, and low MPE (e.g, 808 nm, 0.33 W/cm^2^) in anti-bacterial infections. PTAs of NIR-II PTT relying on the irradiation of NIR-II light exhibit less energy dissipation and advanced tissue penetration depth (nearly 1 cm) [60], thus facilitating the application of PTT in deep bacterial infections. In addition, NIR-II light has a lower photon energy and higher MPE (e.g., 1064 nm, 1 W/cm^2^), allowing for the use of larger power during irradiation without damaging normal tissue [36,61,62].

Currently, the PTAs of NIR-II PTT in antibacterial trials are mainly inorganic, encompassing noble metal materials, metal sulfides, carbon-based nanomaterials, and transition metal oxides because of their controllable structures, highly tunable physicochemical characteristics, and size-dependent optical properties [63]. Organic materials, including small molecules and semiconductor polymers, are also utilized as NIR-II PTAs due to their excellent biocompatibility. Table 1 provides a detailed overview of the PTAs employed in NIR-II PTT for wound infections by bacteria.

**Table 1:** Photothermal agents (PTAs) employed in NIR-II PTT for wound infections by drug-resistant bacteria.

	Advantages	Limitations	PTAs	Laser (nm)	PCE, *η*	Minimum bactericidal concentration	Bacterial killing rate	Wound healing area	Ref
Noble metal nanoparticles	① Strong light absorption, ② Excellent biocompatibility, ③ Tunable size, shape.	① High cost, ② Biotoxicity risk, ③ Light penetration limitation.	Au/Ag NRs	1064	28.80 %	100 μM	79.10 %	100 %	[64]
			ILGA	1064	44.20 %	150 μg/mL	/	83 %	[65]
			Au-CMS NSs	1064	37.79 %	50 μg/mL	100 %	90 %	[66]
			Pt@V_2_C	1064	59.60 %	100 mg/L	99.90 %	99.20 %	[27]
Metal sulfides	① Strong light absorption, ② Easy to synthesize with tunable properties, ③ Lower cost compared to noble metals.	① Potential toxicity, ② Poor chemical stability, *in vivo*.	Cu_9_S_8_ NPs	1060	59.80 %	50 μg/mL	97.90 %	/	[61]
			CuS Nanoflowers	1064	37.60 %	84.9 μg/mL	>90 %	/	[67]
			MoWS_2_	1064	36.90 %	100 μg/mL	99 %	75 %	[35]
			Cu_2_MoS_4_	1064	37.80 %	40 μg/mL	99.90 %	95 %	[68]
			CuS-CaO_2_-Dex	1064	16.80 %	150 μg/mL	98.20 %	82.80 %	[69]
Carbon-based nanomaterials	① High conductivity and stability, ② Low cost and scalability.	① Potential accumulation in the body.	Graphene quantum dots (GQDs)	1064	50.40 %	200 mg/mL	>97 %	/	[44]
			Cu/Mn-DSAzymes	1064	38.70 %	80 mg/mL	100 %	90 %	[70]
Transition metal oxide	① Good PCE, ② High chemical and thermal stability, ③ Good biocompatibility.	① Lower light absorption, ② Complex synthesis processes.	W/Mo-based polyoxometalate (POM)	1060	46.90 %	500 μg/mL	98.00 %	100 %	[71]
			Rough C-Fe_3_O_4_	1064	/	256 μg/mL	99.90 %	/	[62]
			HE MXenes	1064	65.80 %	100 μg/mL	96.50 %	80 %	[72]
			Nb_2_C/Gel	1064	45.55 %	25 mg/mL	92.48 %	/	[73]
Organic small molecules	① High tunability, ② Low toxicity, ③ Simple synthesis.	① Lower PCE, ② Poor stability *in vivo*.	NIR-II xanthene dyes (CNs)	1064	39 %	20 μg/mL	99.40 %	>90 %	[74]
			BTFB @Fe@Van	1064	28.40 %	0.1 mg/mL	98.20 %	98.19 %	[75]
Organic SPNs	① Good biocompatibility and surface modification, ② High tunability in terms of structure.	① Lower chemical stability, ② Complex synthesis process.	Polypyrrole	1064	51.59 %	40 μg/mL	100 %	/	[76]
			pPCP nanoparticles	1064	51.50 %	50 μg/mL	100 %	100 %	[77]
			NP^M123/Fc^	1064	37.80 %	100 μg/mL	90 %	>90 %	[45]
			photothermal polymer	1064	/	75 μg/mL	99.99 %	>90 %	[78]

#### 2.3.1 Noble metal nanoparticles

Au NPs have been the most studied noble metal materials in recent years due to their excellent biocompatibility, simple preparation, and outstanding stability. They exhibit remarkable optical properties, with strong scattering and absorption in the NIR region, making them widely used in PTT [79]. Au NPs exist in various morphologies, such as nanospheres, nanoshells, nanorods, nanorings, and nanostars, depending on different preparation protocols [[80], [81], [82]].

**Fig. 5:**
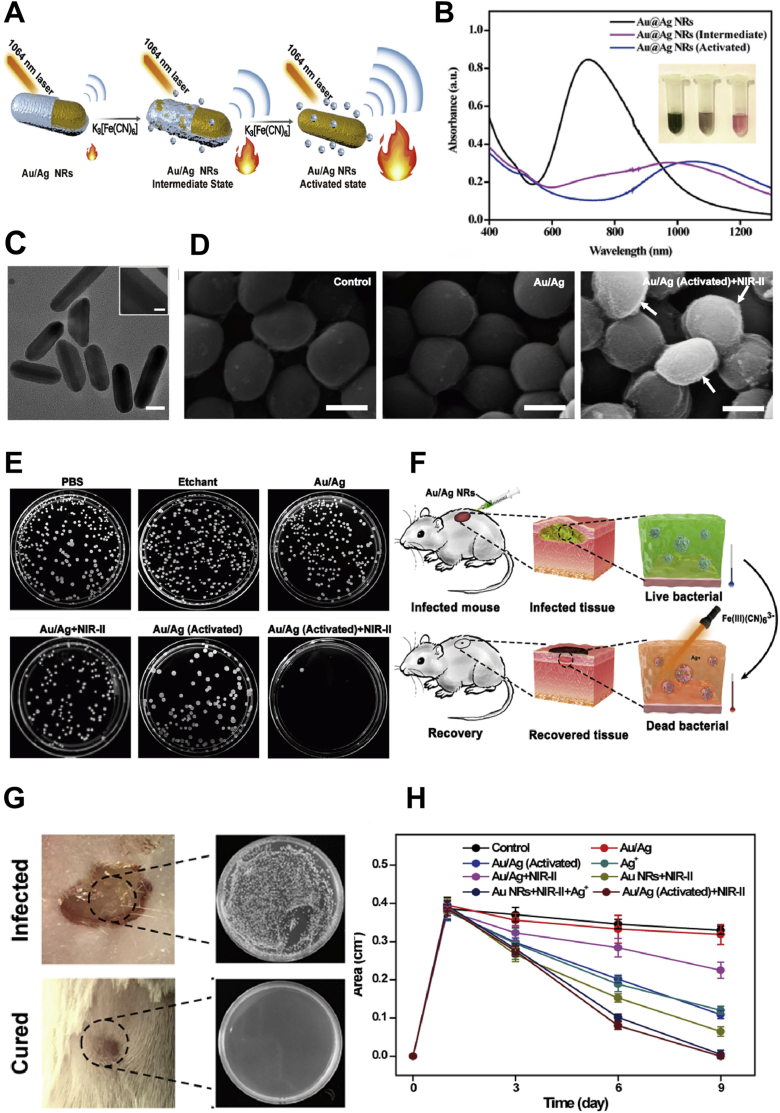
NIR-II photothermal therapeutic effects of Au/Ag core/shell nanorods (NRs) for treating MRSA infections and promoting wound healing. (A) Activatable NIR-II photothermal and photoacoustic properties of Au/Ag core/shell NRs. (B) Uv–Vis–NIR absorption spectra of Au/Ag NRs, Au/Ag NRs (Intermediate), Au/Ag NRs (Activated). (Insert: photograph of the corresponding samples). (C) TEM images of Au/Ag core/shell NRs, scale bar: 20 nm. The insert shows the clear core/shell structure of the corresponding NRs, scale bar: 5 nm. (D) Scanning electron microscopy (SEM) images of MRSA treated with PBS, Au/Ag, and Au/Ag + NIR-II laser, scale bar: 500 nm. White arrows indicate the wrinkled surface of MRSA after synergistic therapy. (E) Representative photos of MRSA colonies on agar plates after different treatments. (F) Schematic illustration of Au/Ag NRs for treating mice with infected wounds. (G) The counting Forming Unit of MRSA was harvested from the cured tissue and infected tissue. (H) Quantitative curves of wound area over time for each treatment group. Reproduced with permission [64].Copyright 2020, Elsevier.

Pan et al. developed a NIR-II-responsive gold nano-hybrid as an effective antibacterial agent. Imipenem (an FDA-approved antibiotic) was loaded into liposomal vesicles, which were then encapsulated in gold nanospheres. Under NIR-II light (1064 nm) irradiation, the gold nanoshell and liposomes ruptured to release encapsulated imipenem for antibacterial therapy. The PCE was 44.2 %, capable of effectively eliminating MRSA *in vitro*, with an 83 % healing rate of the mouse *in vitro* infected wounds after 11 days of treatment. Additionally, the PTAs incorporated into the thermosensitive hydrogel effectively cover the wound. Under NIR-II light exposure, the hydrogel solidifies to promote hemostasis, while also demonstrating antibacterial effects and reducing inflammation, which accelerates wound healing. The gel layer further acts as a physical barrier to prevent bacterial reinfection and creates a moist environment conducive to healing [65]. Mei et al. synthesized miniature Au/Ag nanorods based on Au NPs, which achieved a PTT antibacterial effect under 1064 nm laser irradiation (Fig. 5A). The particles exhibit strong absorption in the NIR-II region (Fig. 5B), and they have a relatively small size (Fig. 5C) and excellent PCE. *In vitro* antibacterial experiments performed with these particles demonstrated excellent antibacterial activity (Fig. 5D). Au/Ag nanorods under the irradiation of NIR-II light eradicated 100 % MRSA (Fig. 5E). Treatment with them for 9 days resulted in the complete healing of infected wounds in a mouse model (Fig. 5F, G, H) [64].

The diabetic wound microenvironment is widely known for its characteristics of high blood sugar, hypoxia, excessive oxidative stress, and bacterial infection, posing challenges for healing. Severe diabetic wounds can result in deep tissue abscesses, gangrene, and infection, eventually damaging deeper structures such as tendons, joints, and bones [83]. In this context, the deeper tissue penetration of NIR-II phototherapy could offer a more effective therapy for diabetic wounds. Shan et al. utilized gold nanoparticles combined with CuCl and other materials to prepare nanomaterials containing glucose oxidase and catalase. Under 1064 nm laser irradiation, the photothermal effect destroyed MRSA bacterial membranes. In deep tissues, achieving a 94 % *in vitro* sterilization rate. Leveraging these enzymatic activities significantly improved the hypoxic and hyperglycemic microenvironment of diabetic wounds. The healing rate of mouse diabetic MRSA-infected wounds after 9 days of treatment reached 90 %, offering a novel direction for diabetic wound treatment [66].

Encouragingly, gold nanomaterials have already been reported for clinical applications. Rastinehad et al. intravenously injected Gold Nanoshells (GSNs) (7.5 mL/kg) into 16 prostate cancer patients, followed by perineal puncture to deliver the treatment light source into the tumors. They then applied an 810 nm laser (4.5–6.5 W, 3 min) for PTT, with continuous irradiation for two days. The GSNs demonstrated excellent biocompatibility and safety in clinic. Fifteen patients completed the treatment without any serious adverse events. Moreover, there were no significant changes in urinary function or sexual health after treatment. At 12 months of follow-up, 87.5 % of the treated areas showed no tumor recurrence [84]. PTT has been used clinically in treating oncology, and with the deeper penetration of NIR-II light, it shows greater promise for future clinical applications, including the treatment of drug-resistant bacterial wound infections.

#### 2.3.2 Metal sulfide

Metal sulfides not only possess merits comparable to those of noble metals mentioned above but also have the advantages of low cost and low tissue toxicity. For example, CuS has strong absorption in the NIR region and outstanding PCE [85] due to the d-d band transition of Cu^2+^ [86]. CuS possesses excellent antibacterial activity, which remains unaffected even when doped with other elements. Furthermore, Cu^2+^ is a crucial trace element for human beings, playing an essential role in promoting wound healing [87]. On the other hand, MoS_2_ nanoparticles, reinforced by strong Mo-S covalent bonds, endow them with remarkable stability. Their advantages, including good PCE and minimal adverse effects, facilitate their wide applications in treating bacterial infections [88].

**Fig. 6:**
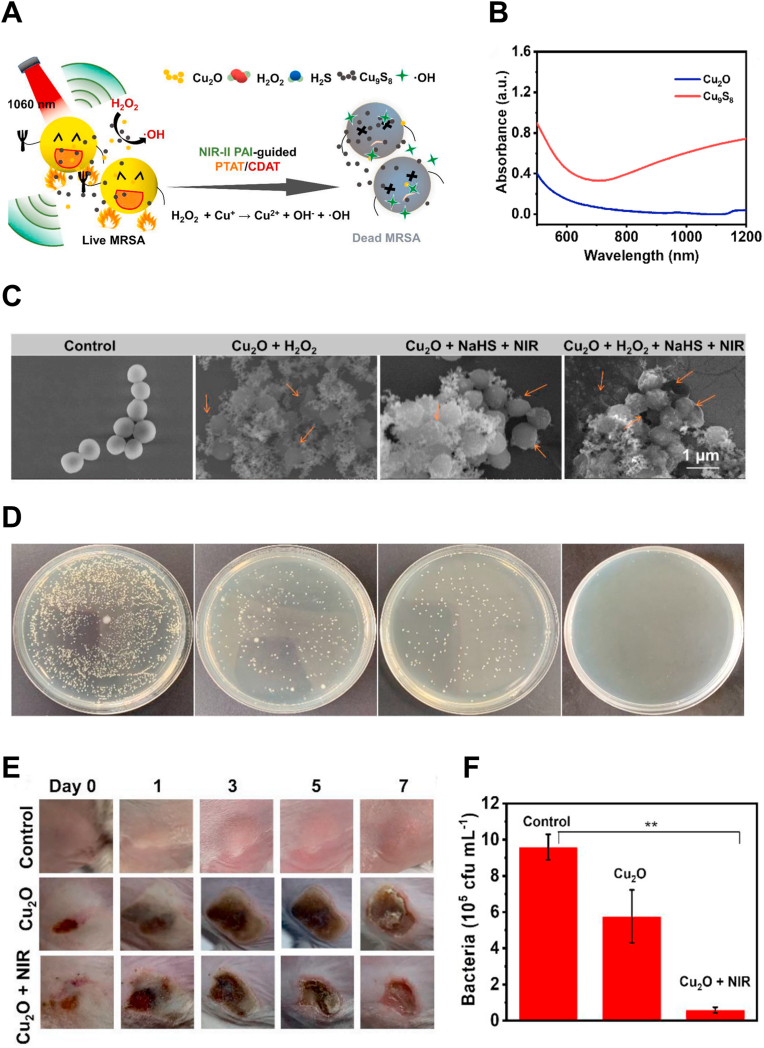
NIR-II photothermal therapy with infection microenvironment-activated Cu_2_O nanoparticles for the treatment of wound infections and the promotion of wound healing. (A) Scheme for the infection microenvironment-activated Cu_2_O nanoparticles for NIR-II photoacoustic imaging-guided photothermal/chemodynamic synergistic anti-infective therapy. (B) UV–Vis–NIR absorption spectra of Cu_2_O NPs and Cu_9_S_8_ NPs. (C–D) SEM images and photos of LB agar plates of MRSA after the following treatments: Control, Cu_2_O NPs + H_2_O_2,_ Cu_2_O NPs + NaHS + NIR, and Cu_2_O NPs + NaHS + NIR + H_2_O_2_. (E) Representative skin images after different treatments. (F) Quantitative analysis of bacterial colonies of day 7 in (E). ∗Indicates significant difference (∗∗*p* < 0.01). Reproduced with permission [61]. Copyright 2021, Elsevier.

Yang et al. synthesized Cu_9_S_8_ nanoparticles (Fig. 6A), which extended their absorption to the NIR-II range (Fig. 6B), with a PCE of up to 59.8 % and strong photostability. Besides their antibacterial activity derived from PTT, these nanoparticles also catalyzed H_2_O_2_, which was rich in the inflammatory condition, producing a synergetic antibacterial effect by generating ROS. This led to bacterial cell membrane damage (Fig. 6C) and finally eliminated 97.9 % of MRSA bacteria (Fig. 6D). *In vivo* experiments further demonstrated that this material could clear bacteria and promote the healing of infected wounds (Fig. 6E and F) [61].

Jiao et al. synthesized a fatty amine-mediated CuS nanoflower structure with variable shapes and sizes. Under 1064 nm laser irradiation (1 W/cm^2^), these nanoflowers heat nearly 50 °C within 10 min, achieving a PCE of 37.6 %. They exhibited broad-spectrum antibacterial activity, with an *in vitro* MRSA clearance rate of over 90 % [67].

Du et al. synthesized tungsten-doped MoS_2_ (MoWS_2_) nanosheets using a hydrothermal method, which exhibited excellent photothermal stability, biocompatibility, and a relatively high PCE of 36.9 %. Under 1064 nm (1 W/cm^2^) laser irradiation for 6 min, 99 % of MRSA were killed *in vitro*. In a mouse wound infection model, it accelerated healing, achieving a healing rate of 75 % after 7 days [35].

#### 2.3.3 Carbon-based nanomaterials

Carbon-based nanomaterials, including graphene, graphene oxide, carbon nanotubes, and carbon quantum dots. Notably, these materials demonstrate strong inherent absorption within the transparent window of biological tissues (750–1700 nm), making them highly suitable for applications in NIR-II PTT [40]. Given their valuable properties—such as excellent chemical stability, biocompatibility, low toxicity to tissues, and the ability to be functionally modified at the surface, carbon-based nanomaterials were widely used in PTT [63]. In 2015, hollow carbon nanospheres with ideal drug-loading capabilities were developed. These spheres delivered therapeutics for PTT while simultaneously generating free radicals, effectively reducing bacterial resistance [89].

**Fig. 7:**
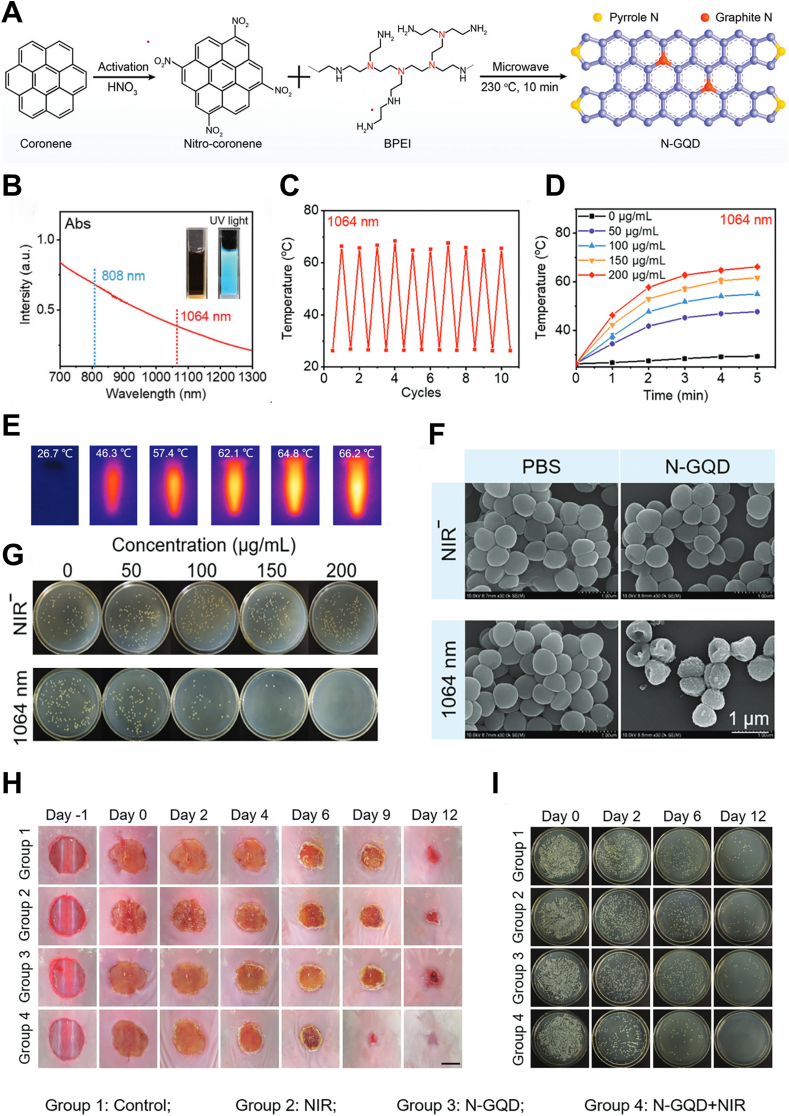
NIR-II photothermal properties of N-GQDs for treating wound infections and promoting healing. (A) Schematic illustration of the synthesis procedures of N-GQDs. (B) NIR absorption of the N-GQDs. (C) Temperature variation of the N-GQDs over ten cycles of laser irradiation. (D) Temperature elevation of the N-GQDs (0, 50, 100, 150, and 200 μg/ml) under 1064 nm (1 W/cm^2^) laser irradiation. (E) Infrared thermographic images of the N-GQDs under continuous 1064 nm laser irradiation. (F) Live/dead SEM images of MRSA in each group. (G) Representative culture images of colonies of MRSA after receiving treatments with various concentrations of N-GQD aqueous solution without or with laser irradiation. (H) Photographs of MRSA-infected skin on days 0, 2, 4, 6, 9, and 12 after different treatments. (I) Photographs of bacterial colonies from the tissues in (H) Reproduced with permission [44]. Copyright 2022, Royal Society of Chemistry.

Graphene quantum dots (GQDs) are nanoscale derivatives of graphene (Fig. 7A) and exhibit absorption in the NIR-II region (Fig. 7B). Geng et al. utilized them as PTAs against bacterial infection with a PCE of 50.4 % under 1064 nm laser, demonstrating strong photostability (Fig. 7C), and remarkable photothermal effects (Fig. 7D and E). They exhibited broad-spectrum antibacterial activity, nearly eradicating multidrug-resistant strains (Fig. 7F and G), eliminating biofilms, and accelerating the healing of infected wounds (Fig. 7H and I) [44].

Qu et al. immobilized Cu and manganese (Mn) single atoms onto carbon nanosheets to prepare Cu/Mn-DSAzymes for PTT bactericidal applications. Under 1064 nm (1.0 W/cm^2^) laser irradiation, the PCE was 38.7 %, enhancing the nanozyme's enzyme activity with 100 % killing rates for MRSA and *E. coli*. *In vivo* mouse wound models showed about 90 % healing of MRSA-infected wounds by day 9, significantly better than the control group [70].

#### 2.3.4 Transition metal oxides

Transition metals (Fe, W, Ti, Mo, etc.) have garnered increasing interest in NIR-II PTT due to their unique properties, such as strong localized surface plasmon resonance (LSPR), tunable broad NIR absorption, and high PCE [90].

**Fig. 8:**
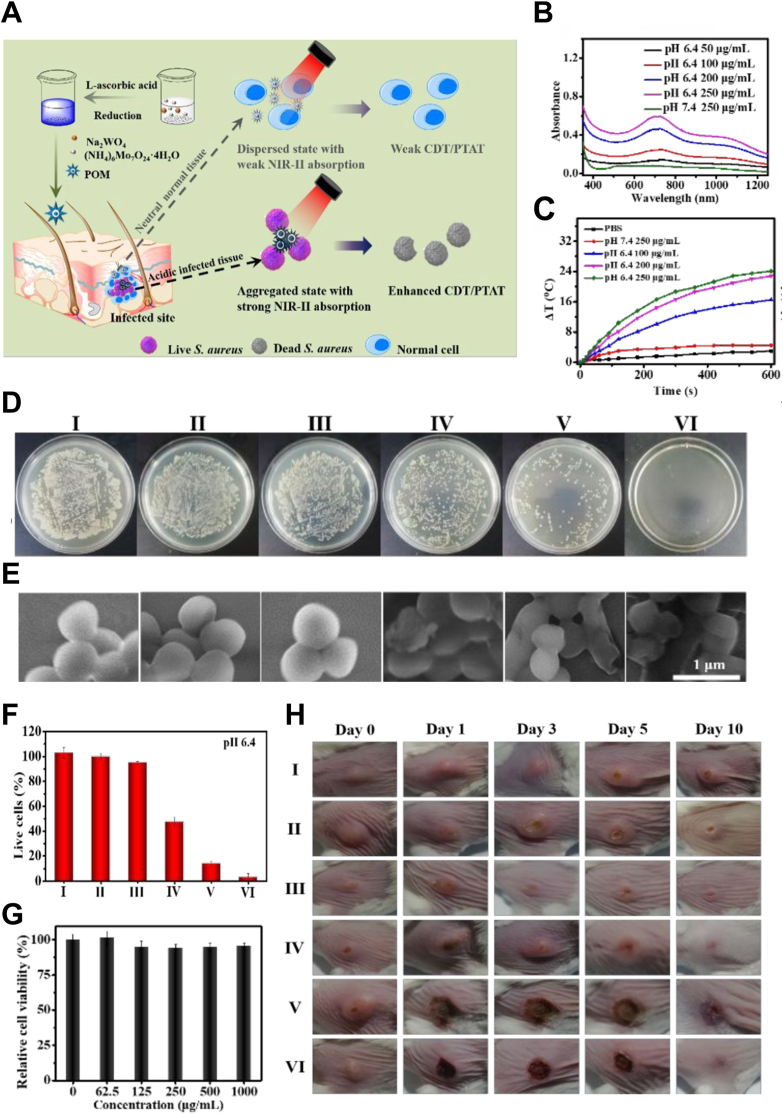
An acidity-responsive polyoxometalate with inflammatory retention for NIR-II photothermal-enhanced chemodynamic antibacterial therapy. (A) Preparation of acidity-aggregated POM clusters for photothermal-enhanced CDT in the NIR-II window. (B) Absorbance spectra of POM solutions at various concentrations or pH values. (C) Photothermal performance of POM with different pH and concentrations under 1060 nm laser irradiation (1 W/cm^2^). (D) Antibacterial effect of POM under different conditions. (I) PBS, (II) H_2_O_2_, (III) POM, (IV) POM + H_2_O_2_, (V) POM + NIR, (VI) POM + H_2_O_2_ + NIR. Scar bar: 4.5 cm. (E) SEM images of drug-resistant *S. aureus* after receiving different treatments. (I) PBS, (II) H_2_O_2_, (III) POM, (IV) POM + H_2_O_2_, (V) POM + NIR, (VI) POM + H_2_O_2_ + NIR. (F) Live/dead cell assay using AM and PI double staining after receiving different treatments of drug-resistant *S. aureus*. (G) Relative cell viability of HUVEC incubated with different concentrations of POM. (H) *In vivo* abscess treatment images after being treated with (I) PBS, (II) H_2_O_2_, (III) POM, (IV) POM + H_2_O_2_, (V) POM + NIR, (VI) POM + H_2_O_2_ + NIR, respectively [71]. Copyright 2021, American Chemical Society.

For example, metal oxides based on W/Mo have been employed for antibacterial purposes. Shi et al. developed a polyoxometalate (POM) based on W and Mo (Fig. 8A), which exhibits absorbance in the NIR-II region (Fig. 8B) for PTT-mediated antibacterial action (Fig. 8C). Under 1060 nm laser irradiation, the heat can not only kill drug-resistant *S. aureus* but also convert hydrogen peroxide at the infection site into ·OH, enhancing chemo-dynamic therapy (CDT) performance and antibacterial efficacy (Fig. 8D, E, F). Meanwhile, these synthetic nano agents exhibited high PCE (46.9 %) and satisfactory biocompatibility (Fig. 8G), showing great potential for treating wound infections in mice (Fig. 8H) [71].

Liu et al. prepared carbon-silicon nanoshells with rough surfaces and uniformly distributed Fe_3_O_4_ nanoparticles with diameters of 6–8 nm around the nanoshells using a simple hetero-aggregation strategy. Fe_3_O_4_ nanoparticles exhibit catalase activity, catalyzing low concentrations of H_2_O_2_ to produce toxic ·OH. These molecules had a photothermal effect and extremely low cytotoxicity. Under NIR-II laser irradiation, a concentration of 128 μg/ml could kill 99.9 % of *E. coli* and 65.7 % of *S. aureus*. *In vivo* experiments have demonstrated that this molecule effectively killed MRSA and promoted wound healing [62].

#### 2.3.5 Organic small molecules

Organic small molecules are attracting attention in the applications of NIR-II PTT, considering their good biodegradability and safety *in vivo*. Additionally, the chemical structure of these small molecules can be finely tuned, leading to structural diversity and widespread utilization in PTT [91].

**Fig. 9:**
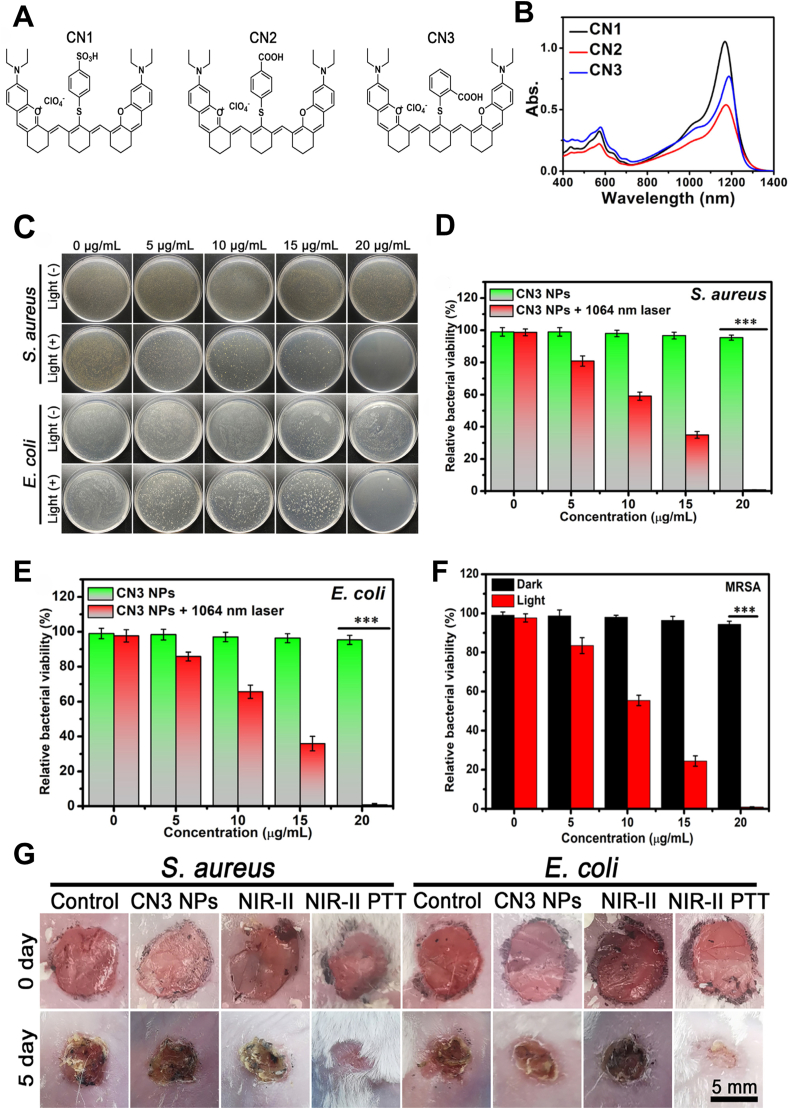
NIR-II photothermal properties of CNs nanoparticles for treating wound infections and promoting wound healing. (A) Chemical structure of CNs. (B) Absorption of CNs in CHCl_3_. (C) Photos of bacterial colonies and bacterial viability of (D) *S. aureus*, (E) *E. coli* treated with CN_3_ NPs (5∼20 μg/mL) in the dark and under 1064 nm laser irradiation (1.0 W/cm^2^, 10 min). (F) Bacterial viability of MRSA treated with CN_3_ NPs (5–20 μg/mL) in the dark and under 1064 nm laser irradiation (1.0 W/cm^2^, 10 min). (G) Photos of the infected wound within 5 days. Reproduced with permission [74]. Copyright 2023, Elsevier.

Zhang et al. developed three new NIR-II xanthene derivatives (CNs) (Fig. 9A), which exhibited strong light harvesting ability around 1180 nm (Fig. 9B). These CNs also demonstrated excellent photothermal stability and high PCE. Specifically, CN_3_, with a higher positive charge, exhibited enhanced antibacterial effects, with photothermal antibacterial activities against *S. aureus* and *E. coli* reaching 99.4 % and 99.2 %, respectively, and the MRSA killing rate approaching 100 % (Fig. 9C–F). Moreover, in mouse infection wound models, the *in vivo* treatment for 5 days resulted in a wound healing rate of over 90 % (Fig. 9G) [74].

A water-soluble organic small molecule, BTFPBA modified with phenylboronic acid (PBA), was assembled with an antibacterial drug vancomycin (Van) and Fe^2+^ to form the BTFB@Fe@Van nano-platform. The Fe^2+^ ions, catalyzing the Fenton reaction with endogenously overexpressed H_2_O_2_ in the microenvironment, generated ·OH, thereby triggering CDT, while vancomycin enhanced the bactericidal effect. Under 1064 nm laser irradiation, these nanoparticles exhibited promising NIR-II photothermal effects, boasting a PCE of 28.4 %, surpassing that of most traditional PTAs. In the mouse model of wound infections, BTFB@Fe@Van cleared 98 % of *S. aureus* and eradicated drug-resistant biofilms [75].

Organic small molecules have high biocompatibility in the body, and the small molecule Indocyanine Green (ICG) has already been reported for clinical treatment of breast cancer. Li et al. injected ICG locally into the lesions of 8 breast cancer patients, followed by *in vitro* irradiation with an 805 nm laser (1 W/cm^2^, 10 min) for PTT. Subsequently, glycated chitosan was injected as an immune adjuvant to further enhance the anti-tumor immune response. The adverse reactions were mainly limited to the treatment area, manifesting as redness, swelling, pain, and ulcers, most of which subsided within 48 h. The clinically beneficial response rate was 75 % [92]. This demonstrates the high safety of small organic molecules in clinical applications.

#### 2.3.6 Organic semiconductor polymer nanoparticles

SPNs offer advantages over existing inorganic nanomaterials, including lower toxicity and stronger biocompatibility. SPNs possess longer absorption wavelengths that extend into the NIR-II region, along with higher absorption capacity and photostability, making them highly promising for biomedical applications. Furthermore, under NIR light irradiation, these SPNs could generate localized heat and reactive oxygen species, synergistically enhancing photothermal therapy for wound infections [[93], [94], [95]].

Polypyrrole (PPy) is widely utilized for the development of materials in both organic electronics and biomedicine due to its high conductivity and photostability. Yang et al. synthesized a photothermal nanoantibiotic (PTNA) as a PTA against bacteria based on PPy, wherein pyrrole was polymerized onto anionic vesicles, integrating PPy's cationic properties. The molecule exhibited a PCE of 51.59 % via the irradiation of a 1064 nm laser, significantly inhibiting *Salmonella typhimurium* growth, alleviating inflammation, and preventing biofilm formation *in vitro* [76].

Zhou et al. prepared a degradable pseudo-conjugated polymer (PCP), with quaternary ammonium cations selectively anchoring and disrupting bacterial membranes through electrostatic interactions, exhibiting photothermal sterilization under 1064 nm laser irradiation, while the molecule is easily decomposed by ROS at the infected site, ensuring high safety. *In vitro* results demonstrated that both multidrug-resistant *S. aureus* and *E. coli* were almost completely removed by receiving the PCP, and after 8 days of *in vivo* treatment, the wounds of infected mice were completely healed [77].

Wu et al. developed an organic polymer composed of amphiphilic polymers and photothermal polymers, which self-assemble to effectively penetrate bacterial biofilms. Upon exposure to 1064 nm laser irradiation, the photothermal effect efficiently disrupts MRSA biofilms and kills nearly all bacteria. Furthermore, it significantly promotes wound healing in mice, achieving a healing rate greater than 90 % by day 9. The molecule also exhibits excellent biocompatibility [78].

### 2.4 The challenges and future directions of NIR-II PTT

Despite the excellent experimental results of NIR-II phototherapy in treating wound infections, several limitations remain to be addressed.

First, the penetration depth of PTT has recently increased, making it difficult to treat wound infections in deep tissues. Studies have shown that increasing the laser spot size can improve tissue penetration. For example, Ash et al. found that when the spot diameter increased from 1 mm to 10 mm, the penetration depth doubled to 10 mm [96]. Tuchin et al. suggested that using high refractive index substances (such as glycerol, glucose, and polyethylene glycol) could reduce light scattering within tissues, enhancing penetration depth [97]. Recent research has shown that with these substances, NIR-II light penetration significantly increased, with light transmittance through pig skin rising from 35 % to 70 % [98]. Additionally, the Silicon-RosIndolizine (SiRos) dyes developed by Meador et al., with longer emission wavelengths, could further improve tissue penetration, offering promising prospects for future phototherapy [99]. Optical waveguides direct light of specific wavelengths to a target, allowing light to travel over long distances without significant attenuation. This overcomes the penetration depth limitations of traditional phototherapy, enabling effective treatment of deep tissue infections while minimizing the impact on surrounding healthy tissue. With the use of biodegradable materials such as polylactic acid, polyglycolic acid, poly lactic-co-glycolic acid, and silk fibroin, the biocompatibility of optical waveguides has been further improved [52]. NIR-II phototherapy offers deeper tissue penetration, and the combination of NIR-II phototherapy with optical waveguides may represent an important direction for treating deep infections.

Secondly, PTT lacks specificity for most bacteria, reducing its antibacterial efficacy and posing risks to healthy tissues. To address this, three targeted strategies have been developed [100]: (1) Conjugation with antibiotics such as vancomycin to target Gram-positive bacteria. Wang et al. used gold nanoparticles (GNPs) modified with vancomycin, which bound to the D-Ala-D-Ala motif in bacterial cell walls, allowing the GNPs to aggregate at infection sites and enhance photothermal effects. In bacteria-free environments, the GNPs remained dispersed, minimizing damage to normal tissues [101]. This method effectively kills resistant bacteria, offers high biocompatibility, and enables simultaneous imaging to monitor treatment, enhancing the bactericidal effect. However, it may be ineffective against Gram-negative bacteria due to the absence of the D-Ala-D-Ala motif in their cell walls. Additionally, the complex preparation of nanoparticles increases production costs and complicates clinical translation. (2) pH-sensitive surface charge switching. Bacterial infection sites are often acidic, and in such environments, certain PTAs (e.g., glycol chitosan derivatives) become positively charged. Since bacterial membranes are typically negatively charged, this electrostatic attraction promotes selective binding of PTAs to bacteria. Korupalli et al. developed a pH-responsive nanoparticle system (PANI-GCS) that targeted acidic abscesses and was activated by NIR light for bacterial killing [22]. The method is an innovative bacterial targeting approach that tracks and kills pathogenic bacteria through tight charge binding, while avoiding damage to normal cells. However, it relies on an acidic microenvironment, and given the complexity of the local infection environment, its ability to consistently kill bacteria requires further investigation. (3) Enzyme-responsive targeting. Bacteria secrete enzymes that degrade protective layers on nanomaterials, exposing active surfaces that adhere to bacterial cells for targeted killing. Liu et al. designed the AA@Ru@HA-MoS_2_ system, which responded to hyaluronidase secreted by bacteria, releasing antimicrobial agents and generating ·OH via MoS_2_ catalysis to kill bacteria [102].

Third, the microenvironment of wound infections is complex, characterized by local high temperature, weak acidity, low oxygen, and overexpression of H_2_O_2_ and H_2_S, which increases the difficulty of treatment [61]. Currently, most antibacterial materials are passive and cannot respond to the wound microenvironment, leading to low utilization of bioactive substances and poor therapeutic effects [42]. Therefore, there is an urgent need to design PTAs that can intelligently respond to the infection microenvironment, achieving antibacterial phototherapy while also improving the local microenvironment and accelerating bacterial clearance. Yang et al. synthesized Cu_2_O nanoparticles, which react with H_2_S in the infected area to form Cu_9_S_8_ NPs. Under 1060 nm light irradiation, they could distinguish between infected and normal tissues, achieving photothermal sterilization. Cu_2_O NPs effectively catalyzed the generation of **·**OH from H_2_O_2_, killing 97.9 % of MRSA [61].

Fourth, for certain infections, using PTT alone is challenging to achieve ideal therapeutic outcomes. Thus, combining PTT with other methods can not only enhance efficacy but also reduce the side effects associated with single therapy. Wei et al. reported significant antibacterial effects when combining phototherapy with antibiotics, chemodynamic therapy, and NO/CO [103]. Similarly, Li et al. demonstrated that combining phototherapy with hydrogels overcomes the limitations of traditional antibacterial methods, offering extensive applications [104]. Xie et al. developed a multifunctional wound dressing combining PTT and nitric oxide gas therapy, which effectively killed MRSA and *E. coli*, eliminated biofilms, enhanced local blood circulation and angiogenesis, and accelerated healing of diabetic wounds [105].

Despite the promising results of NIR-II PTT for wound infection treatment, several challenges remain, including limited tissue penetration, lack of targeting specificity, and the complex infection microenvironment. Future advancements in the design of photothermal agents, as well as the integration of complementary therapeutic strategies, are crucial to overcoming these limitations and improving therapeutic efficacy.

## 3 Photodynamic therapy for wound infections

### 3.1 History of photodynamic therapy

In 1900, Oscar Raab reported that the dye acridine had a lethal effect on paramecia when exposed to specific light [106]. Niels Finsen was awarded the Nobel Prize in 1903 for his study on treating skin tuberculosis using 10.13039/100008457UV light, which started modern PDT. In the same year, Herman Von Tappeiner applied white light together with eosin to locally treat skin tumors, initially describing this effect as “Photodynamic Action” [107]. In 1975, T. J. Dougherty and his colleagues from the Roswell Park Cancer Institute reported the first successful case of hematoporphyrin combined with light to cure tumors in mouse and rat models [108]. During the mid-1990s, PDT was first utilized to treat bacterial infections (periodontal disease) [109]. In 2014, the World Health Organization (WHO) issued a warning about the world entering a “post-antibiotic era”, leading to a shift towards alternative antibacterial strategies such as PDT. In recent years, numerous studies have demonstrated the remarkable therapeutic efficiency of PDT in killing pathogens *in vivo*, such as bacteria, fungi, and viruses. Specifically, PDT has been applied to treat various bacterial infections, including diabetic ulcers, osteomyelitis, otitis media, acne, burns, and wounds in clinics [52].

### 3.2 Mechanism of photodynamic therapy

PDT relies on photosensitizers, specific light irradiation, and interaction with tissue oxygen. Initially, photosensitizers are introduced into the target tissue. Upon absorbing light of specific wavelengths, their internal electrons redistribute, transitioning from the ground state (S_0_) to the excited singlet state (S_1_). This S_1_ typically lasts very short, during which photosensitizers have a higher energy level. Consequently, these electrons are likely to lose excess energy, returning to S_0_ through the emission of light (i.e., fluorescence) or heat. Through processes of internal conversion (IC) and intersystem crossing (ISC), the photosensitizer in S_1_ can further transition to the excited triplet state (T_1_) [[110], [111], [112]]. Photosensitizers in T_1_ have a longer lifetime, facilitating effective energy or electron exchange with surrounding substrates to generate ROS, including ·OH, H_2_O_2_, O_2_·-, and ^1^O_2_ [113].

In PDT, ROS are primarily produced through two pathways: Type I and Type II reactions. In Type I reactions, the excited triplet state (T_1_) of photosensitizer directly exchanges electrons with cellular substrates, forming radicals such as ·OH, H_2_O_2_, and O_2_·-. Conversely, Type II reactions involve energy transfer between the excited photosensitizer and oxygen molecules (O_2_), leading to the direct production of ^1^O_2_ [112]. It is noteworthy that most PDT used for antibacterials falls under Type II reaction, which indicates a relatively higher dependency on the oxygen content at the infection site [114]. The microenvironment of drug-resistant bacterial infections is typically hypoxic, which reduces the effectiveness of Type II PDT, as it consumes oxygen, further limiting its therapeutic potential [115,116]. In contrast, Type I PDT treats infections by generating **·**OH, H_2_O_2_, and O_2_·-, and can function effectively in hypoxic environments because it does not rely on oxygen. Among these, **·**OH is the most reactive radical, capable of reacting with almost all molecules (e.g., lipids, nucleic acids, and proteins), leading to bacterial damage and death [117,118]. H_2_O_2_ is highly oxidative, and its diffusion between cells is nearly unimpeded by cell membranes. It can damage proteins, lipids, DNA, and other cellular components, causing cellular damage. O_2_·-possesses both reducing and oxidizing activities, reacting with other molecules to produce harmful substances. When accumulated in large quantities, it can damage various organelles, leading to bacterial apoptosis and death [114]. In summary, Type I PDT offers stronger antibacterial potential than Type II PDT, as it can function in hypoxic environments, increasing ROS levels and disrupting bacterial cell structure and function.

**Fig. 10:**
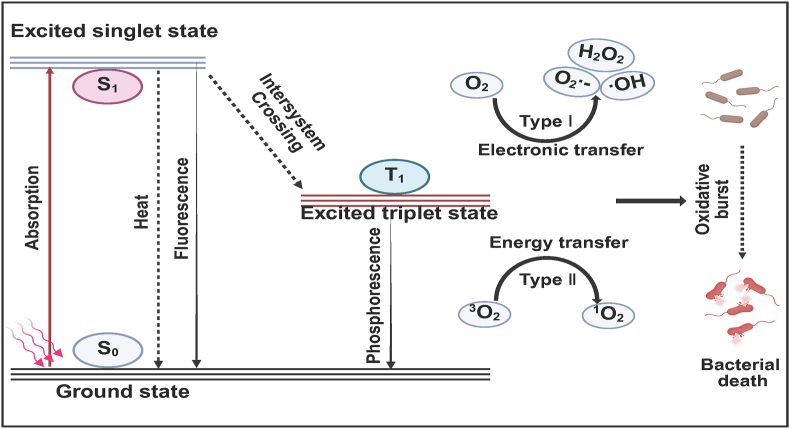
Schematic illustration of the mechanism of antibacterial photodynamic therapy (PDT). S_0_, ground state; S_1_, excited singlet state; T_1_, excited triplet state.

However, the ^1^O_2_ generated by Type II PDT poses a greater threat to bacteria. Its bactericidal mechanism includes direct damage to biomolecules such as unsaturated fats, polypeptides, enzymes, and other cell components, even oxidizing nucleotides, leading to their cleavage, DNA strand breaks, and inhibition of replication [111,119,120]. Fig. 10 illustrates the mechanisms of PDT for antibacterial.

### 3.3 Photosensitizers in NIR-II photodynamic therapy

Initially, photosensitizers were derived from porphyrin structures, with Photofrin® being the first FDA-approved photosensitizer. In 2005, Photofrin® was used for the *ex vivo* treatment of cutaneous *Candida albicans* infection [120]. However, the significant phototoxicity and relatively short excitation wavelength of first-generation photosensitizers limit their clinical application [121,122]. Second-generation photosensitizers, such as phthalocyanines (*Pc*), phenothiazines, and curcumin, offer higher photosensitivity, broader absorption spectra, better tissue selectivity, and faster clearance from the body [123]. To improve the water solubility and therapeutic efficiency of second-generation photosensitizers, nanotechnology has been introduced in the development of third-generation photosensitizers, significantly enhancing their antibacterial activity [124]. Notable third-generation photosensitizers include Metal-Organic Frameworks (MOFs), metal oxides, metal sulfides, carbon-based nanomaterials, organic nanomaterials, and silicon nanoparticles [125,126].

Currently, most of the light sources used in clinical PDT are located in the UV–Vis region, with a wavelength range of approximately 400–670 nm. However, due to the absorption and self-scattering of tissues, the penetration depth of UV–Vis light into tissues is typically only about 0.5 mm–2.5 mm, which limits their applications in treating deep-seated diseases. Additionally, high-intensity light exposure can cause tissue damage at the irradiation site [127]. In contrast, the interaction between light in the NIR-II window and tissues is weak, resulting in low phototoxicity and greater tissue tolerance. This light can penetrate tissues to deeper depths and has a larger MPE value, making it significantly advantageous for treating deep tissue infections [128]. Table 2 shows the photosensitizers used in NIR-II PDT for wound infections by bacteria.

**Table 2:** Photosensitizers used in NIR-II PDT for wound infections by drug-resistant bacteria.

	Advantages	Limitations	Agent	Laser(nm)	Minimum bactericidal concentration	Bacterial kill rate	Wound healing area	Ref
Inorganic nanomaterials	① High stability, ② Effective ROS generation, ③ Tunable properties, ④ Longer tissue penetration.	① Potential toxicity, ② Limited biodegradability.	Ag/BMO NPs	1064	200 μg/mL	99 %	>70 %	[129]
			Ag-doped Au/CdSexSy	1064	50 μg/mL	100 %	/	[130]
Organic nanomaterials	① Good biocompatibility, ② Ease of functionalization, ③ Better biodegradability, ④ Low cost.	① Lower stability, ② Limited ROS generation, ③ Weaker light absorption.	PNIR-II	1064	10.0 μg/mL	99.99 %	100 %	[131]

#### 3.3.1 Inorganic nanomaterials

**Fig. 11:**
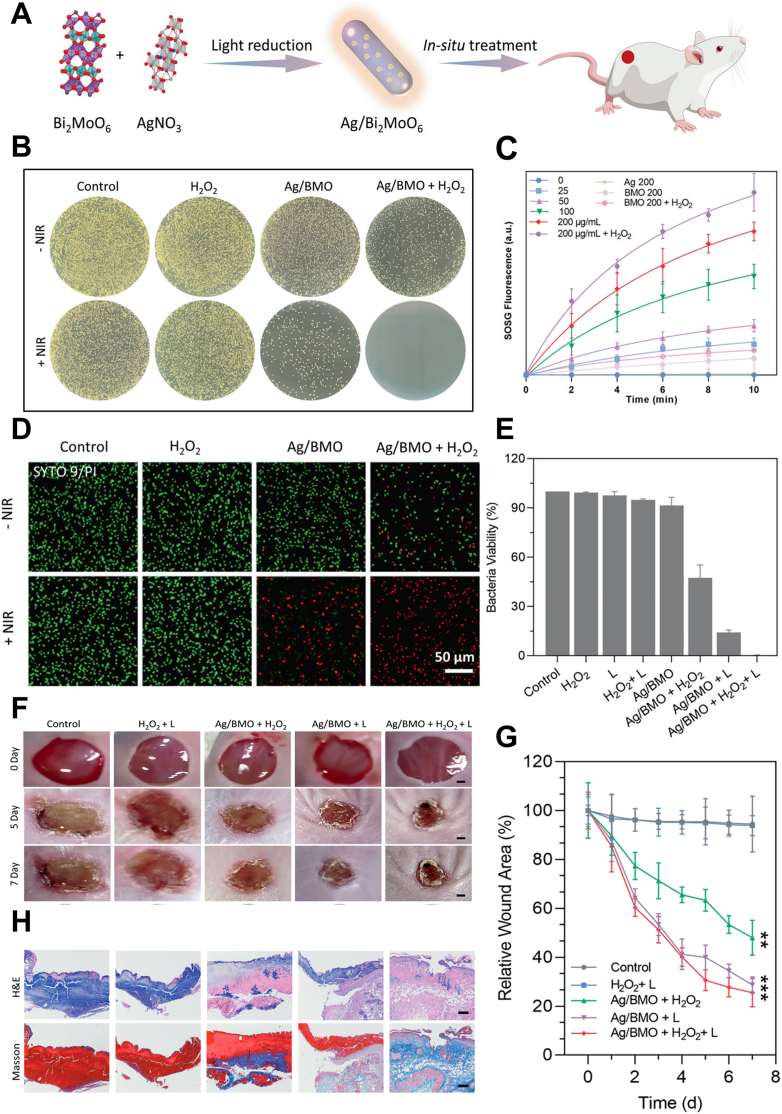
NIR-II photodynamic therapy using Ag/BMO nanozymes to treat wound infections and promote wound healing. (A) Preparation of Ag/BMO nanozyme and NIR-enhanced catalytic activity mechanisms for synergistic bacterial therapy. (B) Plate photographs demonstrating the antibacterial activity of Ag/BMO NPs. (C) The singlet oxygen (^1^O_2_) detection using the SOSG probe. (D) Confocal Laser Scanning Microscopy (CLSM) images of MRSA stained by Live/dead dye following incubation with Ag/BMO NPs with or without NIR laser irradiation. (PI emits red fluorescence and SYTO9 emits green fluorescence). (E) Corresponding quantitative survival ratio of MRSA in (B). (F) Photographs of MRSA-infected wounds in various groups after treatments. (G) The change of wound areas for 7 days. ∗∗*p* < 0.01, ∗∗∗*p* < 0.001. (H) H&E and Masson-stained tissue slices of infected wounds. Reproduced with permission [129]. Copyright 2022, The Author(s).

Cao et al. synthesized Bi_2_MoO_6_ core using Bi and Mo and then introduced Ag to prepare Ag/Bi_2_MoO_6_ nanozymes (Ag/BMO NPs) with enhanced catalytic activity under NIR-II light (Fig. 11A). These nanozymes were designed for synergistic antibacterial therapy, combining nanozyme-enabled catalytic therapy (NCT) and NIR-II PDT. The excellent antibacterial performance of Ag/BMO NPs is attributed to their peroxidase-like activity and strong ROS generation, which enable them to catalyze the generation of ·OH from H_2_O_2_ (Fig. 11C). *In vitro* experiments demonstrated that the nanomaterial achieved a killing rate of up to 99 % against MRSA (Fig. 11B–D, E), with a wound healing area exceeding 70 % in mice after 8 days of treatment (Fig. 11F, G, H) [129].

Metal nanomaterials have garnered significant attention for their potential in antibacterial therapies. Wang et al. synthesized hybrid plasmonic Au/CdSexSy with precise Ag doping (ACA) nanodumbbells, which were designed to achieve ideal NIR-II light-induced antibacterial PDT. Notably, the Ag-doped (ACA) mixed nanodumbbells could generate approximately 40 times more O_2_·- and a higher amount of **·**OH compared to conventional methods. This enhanced generation of ROS is due to the superior electron-hole separation ability and utilization efficiency of the nano dumbbells, which significantly improved PDT efficiency over traditional core-shell structures and coating strategies. *In vitro* antibacterial experiments further demonstrate the efficacy of these nano-dumbbells, showing that they can kill up to 100 % of *S. aureus* and eliminate bacterial biofilms [130].

#### 3.3.2 Organic nanomaterials

Bu et al. developed a novel polymer photosensitizer (PNIR-II) based on NIR-II PDT. This photosensitizer forms a large conjugated structure by alternately linking the electron donor thiophene with the electron acceptors diketopyrrolopyrrole (DPP) and boron dipyrromethene (BODIPY), enabling deep tissue penetration and maintaining 50 % PDT efficacy under a 2.6 cm tissue barrier. Upon irradiation with a 1064 nm laser (1.0 W/cm^2^, 10 min), the nanoparticles not only generated ^1^O_2_ but also reacted with nitric oxide (NO) to produce reactive nitrogen species (RNS) with enhanced bactericidal activity, achieving a 99.99 % kill rate against MRSA while effectively eradicating bacterial biofilms. Ultimately, under the 2.6 cm tissue barrier, the PNIR-II molecule successfully cleared MRSA from mouse wounds via PDT, with a 100 % wound healing rate after 7 days.

Methylene blue (MB) is an organic small molecule with the advantages of low toxicity and low cost. In 2024, Ferreira et al. reported using MB for PDT to treat diabetic foot ulcers in a study involving 21 patients. The wounds were soaked in 0.01 % MB for 5 min, followed by irradiation with a 660 nm laser (1 W/cm^2^, 1 min) once a week, with an average of 8 treatments. After treatment, the wound area significantly reduced, and healing was improved. No notable adverse reactions were observed during the study, demonstrating the good safety profile of MB for clinical applications [132].

### 3.4 Challenges and future directions of NIR-II PDT

Like NIR-II PTT, NIR-II PDT has its limitations in addition to insufficient tissue penetration depth and a lack of bacterial targeting.

First, the design of NIR-II photosensitizers remains a challenging task. Most NIR-II PDT for antibacterial applications belongs to Type II, which relies more on the oxygen content at the infection site [96]. However, local tissues in infectious diseases often exhibit adverse factors such as hypoxia and reduced oxygen concentration, limiting the effectiveness of PDT in these conditions. Therefore, it was particularly important to develop Type I PDT photosensitizers that are oxygen-free for NIR-II PDT. The principles and strategies for designing Type I photosensitizers included donor-acceptor systems, anion-π+ incorporation, polymerization, and cationization [133]. Recently, many Type I photosensitizers were developed, including metals and metal compounds, carbon-based nanomaterials, and organic molecules, which showed good efficacy in treating bacterial infections [114]. For instance, Zhang et al. synthesized TiO_2_, which generated a large amount of ·OH and O_2_·- under 1060 nm light irradiation, achieving a 95.2 % kill rate against *S. aureus* [134].

Secondly, in PDT, excessive production of ROS can lead to uncontrolled oxidative stress, which exacerbates inflammation and delays wound healing [135,136]. Therefore, it is essential to monitor ROS levels in PDT-treated wounds, and developing ROS-responsive materials represents a promising strategy. Selenium (Se), for example, has garnered significant attention due to its high ROS sensitivity. Wang et al. synthesized a gelatin-hyaluronic acid hydrogel embedded with Se-modified cerium dioxide nanoparticles (Gel-HA-Se@CeO_2_ NPs) for treating infected wounds. In addition to generating ROS, Se@CeO_2_ NPs can decompose H_2_O_2_, thereby reducing ROS levels in the wound [137]. Furthermore, hydrogels can be enhanced with various ROS-scavenging materials, including antioxidants, enzymes, and nanomaterials [135]. For instance, 4-octyl itaconate (4OI), a derivative of the metabolite itaconate, possesses anti-inflammatory and antioxidant properties. When 4OI-modified black phosphorus (BP) nanosheets are incorporated into the hydrogel, they improve diabetic wound healing. Under light exposure, 4OI-BP enhances the PDT effect, while in the absence of light, BP acts as a carrier and regulates the controlled release of 4OI, thus mitigating excessive ROS-induced damage to endothelial cells [138]. In conclusion, controlling ROS levels during PDT treatment of wound infections is crucial to alleviate the adverse effects of excessive ROS, thereby promoting wound healing and minimizing both inflammation and oxidative damage.

Third, a single PDT application still yields suboptimal results. It reported that introducing antibiotics based on PDT can yield stronger bactericidal effects against resistant bacteria [139]. We believe that integrating PDT with other methods for the treatment of bacterial infections can not only mitigate the side effects of individual treatments but also enhance overall efficacy.

Lastly, greater attention should be given to the safety management of light therapy during its use in PDT. Since NIR-II light is invisible to the human eye and does not generate heat like PTT, it is less detectable. Therefore, precise control over the light source intensity, exposure duration, irradiated area, and direction is necessary to minimize unnecessary damage. Furthermore, developing wearable or portable NIR-II light source devices for flexible use in clinical settings, while ensuring safety, is one of the future research directions [140].

## 4 Combination of PTT and PDT

Although PTT and PDT have achieved promising results in sterilization, their applications have limitations. Therefore, combination therapies involving both PDT and PTT have been explored in recent years. The combined application has the following advantages.

(1) One approach to this combination is to achieve simultaneous synergistic effects of PTT and PDT using single light irradiation, which simplifies the procedure by eliminating the need for multiple laser irradiations and shortens treatment time [141].
(2) Combining PDT and PTT can reduce drug dosage, minimize the side effects associated with single methods, and enhance bactericidal efficiency [134,142]. Specifically, the heat generated by PTT can reduce bacterial activity, making them more sensitive to ROS. At the same time, the heat increases the permeability of bacterial cell membranes to ROS, thereby reducing the required ROS levels. Similarly, ROS can decrease bacterial heat resistance, thereby lowering the temperature of PTT and minimizing tissue damage [24]. For example, Zha et al. synthesized a bilayered gelatin/acryloyl β-cyclodextrin (BGACD) hydrogel, with the lower layer loaded with humic acids (HAs). This molecule achieved 95 % MRSA eradication through combined phototherapy. The combination of PTT and PDT allowed HAs to induce a lower temperature, preventing damage to normal tissues. Furthermore, the heat facilitated the penetration of ROS into bacteria, while excess ROS near normal tissues could be cleared by HAs to avoid unintended damage [143].
(3) Combined therapy can cause mechanical lysis to kill both drug-sensitive and resistant bacteria, making it a promising strategy to address the issue of antibiotic resistance [144]. Table 3 shows the photosensitizers used in NIR-II PDT + PDT for wound infections by bacteria.

**Table 3:** PTAs**/**Photosensitizers used in NIR-II PTT + PDT for wound infections by drug-resistant bacteria.

Agent	PTT Laser(nm)	PDT Laser(nm)	PCE, *η*	Minimum bactericidal concentration	Bacterial kill rate	Wound healing area	Ref
AuNR-SiO_2_-Cu_7_S_4_	1064	1064	55.30 %	100 μg/mL	98.30 %	97.8 %	[145]
CuS/Qu–CNGs	1064	1064	/	0.2 μg/mL	100 %	100 %	[146]
SnO_2-x_-SiO_2_-HA	1064	1064	31.10 %	250 μg/mL	>90 %	92.30 %	[141]
CuNx-CNS	1064	1064	40.90 %	1 mg/mL	100 %	100 %	[147]

**Fig. 12:**
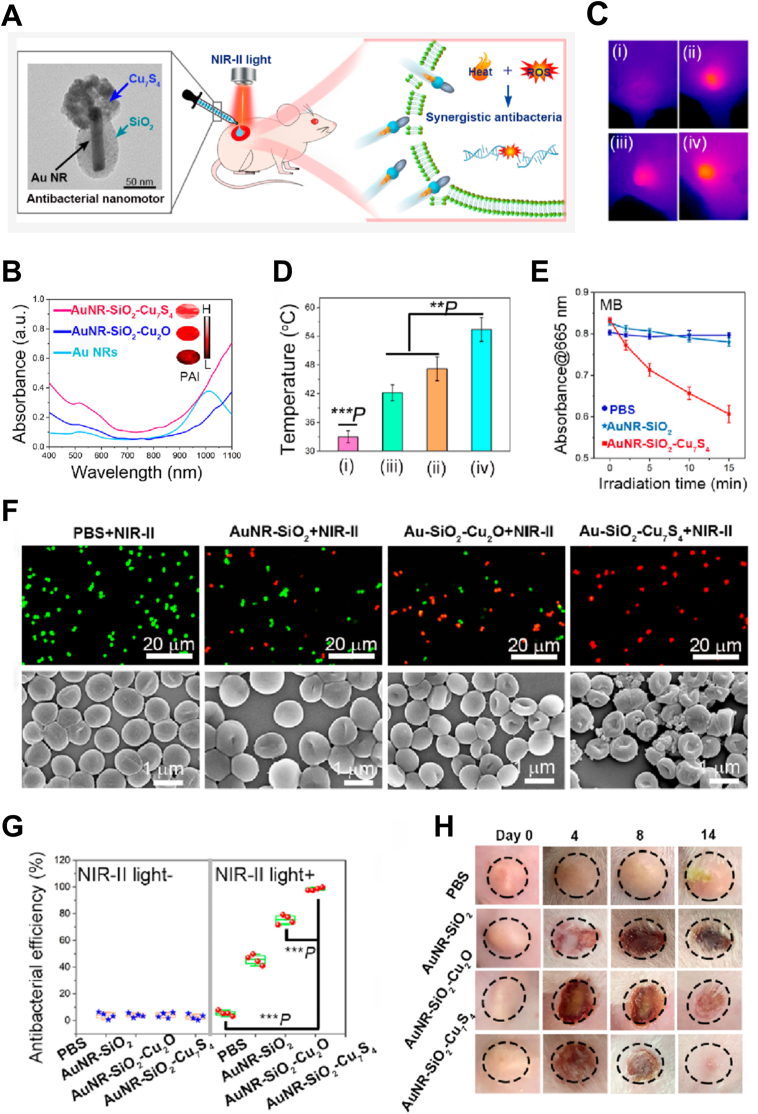
Drug-dye antimicrobial nanomotor for precise treatment of multidrug-resistant bacterial infections. (A)NIR-II light-triggered synchronous autonomous movement and synergistic photothermal/photocatalytic antibacterial of the nanomotors for enhancing transdermal penetration and effectively treating MRSA infections. (B) UV–Vis–NIR absorption spectra of different samples. (C) Thermal images and (D) corresponding temperature changes at the skin abscess site in mice undergoing different treatments (1064 nm,0.75W/cm^2^): (i) PBS, (ii)AuNR-SiO_2_, (iii)AuNR-SiO_2_-Cu_2_O, and (iv) AuNR-SiO_2_-Cu_7_S_4_ groups. (E) ROS generation as detected by methylene blue (MB) upon exposure to 1064 nm light irradiation (0.75W/cm^2^). (F) Live/dead stained images of MRSA and bacterial morphology observed by SEM after different treatments. (G) Antibacterial efficiency of MRSA. (H) Photographs of MRSA-infected skin abscess model mice undergoing different treatments under NIR-II light irradiation. The dashed circle indicates the MRSA-infected skin abscess site. Reproduced with permission [145].Copyright 2023, American Chemical Society.

For example, Liu et al. reported a dual plasmonic nanomotor (AuNR-SiO_2_-Cu_7_S_4_) featuring a unique Janus structure, which facilitates directional motion. This nanomotor with remarkable absorption in the NIR-II region effectively integrated the PTT and PDT (Fig. 12A and B), significantly enhancing its antibacterial performance. Under 1064 nm (0.75 W/cm^2^, 5 min) laser irradiation, in addition to the thermal effect (Fig. 12C and D), it generated a large amount of ·OH (Figs. 12E) and ^1^O_2_, effectively disrupting the bacterial cell membrane and its biological functions. Furthermore, the directional movement of the nanomotor enhances its interaction with bacteria, thereby improving the bactericidal effect. *In vitro*, it eliminates 98.3 % of MRSA (Fig. 12F and G). In a mouse infection model, the nanomotor significantly accelerated wound healing, achieving a healing rate of 97.8 % after 14 days (Fig. 12H) [145].

Nain et al. developed a quercetin-based carbonized nanogel (CuS/Qu–CNGs), which successfully achieved a synergistic antimicrobial effect through combined PTT and PDT. Under 1064 nm (0.94 W/cm^2^, 10min) light irradiation, the molecule exhibited nearly 100 % bactericidal activity against MRSA, with a minimum inhibitory concentration reduced by 125-fold compared to traditional monomeric quercetin, and was able to effectively penetrate and eliminate MRSA biofilms. Additionally, the nanocomposite demonstrated anti-inflammatory effects during wound healing, promoting angiogenesis, epithelialization, and collagen synthesis, thereby accelerating chronic wound healing. After 18 days, the wounds of diabetic mice treated with CuS/Qu–CNGs were completely healed, significantly outperforming the control group [146].

Tin dioxide (SnO_2_) is an important semiconductor material used in the biomedical field with good antibacterial and antioxidant properties [148,149]. Gao et al. developed an innovative nanotheranostic agent based on oxygen-deficient SnO_2_−x nanoparticles, synthesized via high-temperature NaBH_4_ reduction to produce oxygen-deficient SnO_2_−x. Modification with hyaluronic acid (HA) further enhanced the stability and targeting ability of the nanoparticles, resulting in strong absorption in the NIR-II window. This enabled the nanomaterial to efficiently combine PTT and PDT for antibacterial applications. Under 1064 nm (1.0 W/cm^2^, 10 min) light irradiation, the agent achieved over 90 % MRSA eradication efficiency. Furthermore, *in vivo* studies demonstrated that SnO_2_−x@SiO_2_-HA significantly accelerated wound healing, with a 92.3 % wound healing rate in mice after 14 days [141].

The combination of PTT and PDT for antibacterial therapy offers promising results but still faces several limitations, which can be addressed through targeted improvements.

(1) The selection of molecules used in combination therapy is currently limited, and there is a need for further optimization of nanomaterials. One potential improvement is enhancing NIR-II absorption by doping or alloying nanomaterials, which can improve their plasmonic or photonic properties. Additionally, optimizing the size, shape, and composition of nanomaterials can help maximize their absorption cross-section while reducing energy dissipation [150]. For example, Zha et al. doped humic acids (HAs) and astragaloside IV (AS) into a BGACD hydrogel. This hydrogel, when combined with PTT/PDT, effectively kills MRSA in the early stages of infection, followed by the controlled release of HAs and AS. These compounds help reduce ROS levels, promote M2 macrophage polarization, and stimulate angiogenesis, thus enhancing tissue regeneration [143].
(2) Another challenge is the lack of targeting specificity in nanomaterials for certain bacterial strains, which can increase the risk of toxicity. A possible solution is to functionalize nanomaterials with targeting ligands such as antibodies, peptides, or aptamers, which can improve their specificity for pathogenic bacteria. Alternatively, designing pH-, enzyme-, or temperature-sensitive nanoplatforms can ensure that drugs are released or phototherapy is activated only at the infection site [143].
(3) Furthermore, the therapeutic effect can be enhanced by combining other strategies. For example, pairing antibiotics with phototherapy can be highly effective. After phototherapy removes bacterial biofilms, antibiotics can be more effective in killing drug-resistant bacteria, thereby further strengthening the antibacterial effect [24].

## 5 Conclusions and perspectives

PTT and PDT have been applied in the treatment of drug-resistant wound infections by using light to irradiate PTAs or photosensitizers, which further generate heat or ROS to kill bacteria without inducing antibiotic resistance. Our focus is on NIR-II phototherapy, which offers deeper tissue penetration, reduced phototoxicity, and less local tissue damage, along with a higher MPE, making it advantageous for treating deep wound infections.

However, NIR-II phototherapy still faces several key challenges. Firstly, while the tissue penetration depth of phototherapy has significantly improved, it still needs optimization for the treatment of deeper wound infections. Secondly, the lack of bacterial-targeting specificity may cause damage to normal tissues, affecting the therapeutic outcome. Moreover, the complex microenvironment of the infection site (such as hypoxia and high H_2_O_2_ levels) limits the generation and utilization of ROS in PDT, and the use of single therapies is insufficient to meet the treatment needs of complex wound infections.

To address these challenges, future NIR-II PTT and PDT might focus on the following directions: (1) Development of smart materials: design PTAs and photosensitizers that can respond to the infectious microenvironment (such as acidity, hypoxia, and high H_2_O_2_) to improve the local microenvironment. These materials aim to enhance the local microenvironment by promoting the proliferation of M2 macrophages, which secrete anti-inflammatory cytokines (e.g., IL-10, TGF-β) to suppress excessive inflammation. Additionally, M2 macrophages generate VEGF and fibroblast growth factor (FGF), which stimulate the proliferation and migration of endothelial cells, thereby facilitating angiogenesis. This process helps alleviate local hypoxia, accelerates wound healing, and concurrently provides targeted antibacterial effects. (2). Development of innovative photosensitizers: Prioritize the development of Type I PDT photosensitizers that do not rely on oxygen to address the issue of hypoxia at the infection site and enhance therapeutic efficacy. (3). Multi-modal synergistic therapy: Integrate PTT, PDT, and other therapeutic modalities (such as antibiotics, chemodynamic therapy, and hydrogel therapy) to enhance therapeutic efficacy through synergy and reduce side effects of single therapies. (4). Clinical Translation: The clinical translation of NIR-II PTT and PDT for treating complex drug-resistant wound infections, such as diabetic wounds, faces several key challenges. ①Regulatory approval: The approval process for light-based therapies is complex, requiring extensive clinical data testing. ②Nanomaterial production and scalability: Nanomaterial production must focus on reducing costs while maintaining consistent quality. Ensuring large-scale production and biocompatibility is crucial for clinical translation. ③Safety of PTAs and photosensitizers: Safety assessments of PTAs and photosensitizers are crucial, focusing on toxicity, biocompatibility, and clearance, especially for elderly and immunocompromised patients. ④Clinical considerations: Low-cost, low-power, user-friendly NIR-II light source devices are needed for widespread use. Additionally, sprayable or gel-based formulations should be developed to simplify treatment, reduce patient discomfort, and improve patient compliance.

In conclusion, NIR-II PTT and PDT are effective strategies for combating drug-resistant wound infections. Through continuous innovation and optimization, this field is expected to make a leap from basic research to clinical practice, ultimately bringing tangible health benefits to global wound infection patients.

## CRediT authorship contribution statement

**Xiang Chen:** Writing – original draft, Visualization, Validation, Software, Resources, Methodology, Investigation, Formal analysis, Data curation, Conceptualization. **Zhanming Lin:** Writing – original draft, Visualization, Validation, Software, Resources, Methodology, Investigation, Formal analysis. **Nuo Cheng:** Writing – original draft, Visualization, Validation, Software, Resources, Methodology, Investigation, Formal analysis. **Yongjun Mo:** Writing – original draft, Visualization, Validation, Software, Resources, Methodology, Investigation, Formal analysis. **Liang Lu:** Validation, Software, Resources, Methodology, Investigation, Formal analysis, Data curation, Conceptualization. **Jun Hou:** Validation, Resources, Methodology, Investigation, Formal analysis, Data curation, Conceptualization. **Zhenghui Li:** Writing – review & editing, Supervision. **Xinyu Nie:** Writing – review & editing, Supervision, Conceptualization. **Shuai Gao:** Writing – review & editing, Supervision, Funding acquisition, Conceptualization. **Qikai Hua:** Writing – review & editing, Supervision, Funding acquisition, Conceptualization.

## Ethics approval and consent to participate

Ethics approval and consent to participate do not apply to this review manuscript.

## Consent for publication

The authors affirm that they have obtained all appropriate consents for publication from the copyright holders of the images used in this manuscript.

## Availability of data and materials

All data referenced are available in articles cited in this manuscript.

## Fundings

This study was supported by grants from the 10.13039/501100001809National Natural Science Foundation of China (82260448); Guangxi Natural Science Foundation of China (2024GXNSFDA010024); Guangxi Key Research and Development Plan (2021AB11027); 10.13039/501100011416State Key Laboratory of Advanced Optical Communication Systems and Networks, China (2024GZKF13); 10.13039/100000968American Heart Association (23POST1029102).

## Declaration of competing interest

The authors declare that they have no known competing financial interests or personal relationships that could have appeared to influence the work reported in this paper.

## Data Availability

No data was used for the research described in the article.

## Abbreviations

PTT: Photothermal therapy
PDT: Photodynamic therapy
PTAs: Photothermal agents
ROS: Reactive oxygen species
UV–Vis: Ultraviolet–visible light
NIR-I: Near-infrared I
NIR-II: Near-infrared II
MPE: Maximum permissible exposure
PCE: Photothermal conversion efficiency
LSPR: Localized surface plasmon resonance
IL-1: Interleukin-1
IL-6: Interleukin-6
TNF-α: Tumor Necrosis Factor-alpha
EGF: Epidermal growth factor
VEGF: Vascular endothelial growth factor
PDGF: Platelet-derived growth factor
MRSA: Methicillin-resistant *Staphylococcus aureus*
CDT: Chemo-dynamic therapy
NCT: Nanozyme-enabled catalytic therapy
IC: Internal conversion
ISC: Intersystem crossing
MOFs: Metal-Organic Frameworks
